# Some Wildfire Ignition Causes Pose More Risk of Destroying Houses than Others

**DOI:** 10.1371/journal.pone.0162083

**Published:** 2016-09-06

**Authors:** Kathryn M. Collins, Trent D. Penman, Owen F. Price

**Affiliations:** 1 Centre for Environmental Risk Management of Bushfires, University of Wollongong, Wollongong, New South Wales, Australia; 2 School of Ecosystem and Forest Science, University of Melbourne, Creswick, Victoria, Australia; University of Saskatchewan, CANADA

## Abstract

Many houses are at risk of being destroyed by wildfires. While previous studies have improved our understanding of how, when and why houses are destroyed by wildfires, little attention has been given to how these fires started. We compiled a dataset of wildfires that destroyed houses in New South Wales and Victoria and, by comparing against wildfires where no houses were destroyed, investigated the relationship between the distribution of ignition causes for wildfires that did and did not destroy houses. Powerlines, lightning and deliberate ignitions are the main causes of wildfires that destroyed houses. Powerlines were 6 times more common in the wildfires that destroyed houses data than in the wildfires where no houses were destroyed data and lightning was 2 times more common. For deliberate- and powerline-caused wildfires, temperature, wind speed, and forest fire danger index were all significantly higher and relative humidity significantly lower (*P* < 0.05) on the day of ignition for wildfires that destroyed houses compared with wildfires where no houses were destroyed. For all powerline-caused wildfires the first house destroyed always occurred on the day of ignition. In contrast, the first house destroyed was after the day of ignition for 78% of lightning-caused wildfires. Lightning-caused wildfires that destroyed houses were significantly larger (*P* < 0.001) in area than human-caused wildfires that destroyed houses. Our results suggest that targeting fire prevention strategies around ignition causes, such as improving powerline safety and targeted arson reduction programmes, and reducing fire spread may decrease the number of wildfires that destroy houses.

## Introduction

Many people live in areas that place them at risk from the devastating impact of wildfires. There are numerous examples globally of wildfires that have caused the loss of life and destruction of many houses e.g. [1–7]. These events typically cause major social disruption and may result in billions of dollars of damages. For example, the 2009 Black Saturday fires in Victoria impacted on 78 towns and resulted in 173 lives lost, 2133 houses destroyed and direct economic costs conservatively estimated at $4.4 billion [1]. Although relatively few fires cause major losses of human lives and homes [8], there is potential for the number of destructive wildfires to increase due to population growth, more homes being built in the wildland urban interface [9–11] and climate change [12–14].

The probability of a wildfire destroying a house is determined by three elements: the probability of an ignition occurring, the probability of a fire spreading to where a house is located and the probability that a house will be destroyed in that fire [15]. If an ignition occurs, fire suppression may stop a wildfire from spreading and reaching houses although this is dependent on a number of factors such as weather [16–18], fuel type [16], fuel load [17, 19], slope [17, 19], response time [16, 17], number of resources available [19] and the fire size when resources commence suppression activities [16–18]. If fire spreads to where houses are located, the probability of a house being destroyed depends on the level of fire exposure (radiant heat, flame contact and ember density) [20, 21], the vulnerability (construction, design, material and siting) of the house [20–22] and suppression actions of fire agencies or residents [20, 23, 24].

Fire weather is the dominant factor that determines the probability of wildfire destroying a house [25–28]. Fire weather has a major influence on ignition probability [29], fire spread, ember spotting distance and fire intensity [30, 31] which in turn determines the probability of fire suppression success [31–33]. Most houses destroyed by wildfires occur during periods of extreme fire weather [34–36] when opportunities for safe and effective fire suppression actions are very restricted [17, 37]. Under these weather conditions, the effectiveness of fuel reduction treatments is also limited [27, 37–39] but house survival is more likely if the treatments are located in areas adjacent to houses than distant landscape treatments [25, 26, 28, 40–42].

Wildfire ignitions are either due to human, through accidental or deliberate action, or natural sources. The spatial and temporal pattern of ignitions are associated with complex drivers that vary with different ignition causes e.g. [29, 43, 44]. Many human-caused ignitions occur close to roads [29, 44] and populated areas [43, 45, 46] whereas lightning ignitions are more likely to occur away from the wildland urban interface in low population density areas [29, 47]. Ignition location influences the probability of a wildfire impacting on houses. The closer the ignition is to houses, the more likely it will spread to a house under any weather conditions [48]. Under extreme weather conditions, wildfires starting long distances from the wildland urban interface may reach houses [26, 48].

An understanding of which ignition causes result in destroyed houses can provide a valuable insight into identifying potential management strategies to reduce the number of wildfires that destroy houses. As far as we can ascertain, there have been no previous studies comparing the role of ignition cause on destroyed houses. Previous simulation studies have suggested that an increase in ignition management effort, simulated by a reduction in ignition probabilities, can be more effective than fuel management in reducing area burned adjacent to assets [41].

In this study, we investigated the relationship between wildfire ignition causes and destroyed houses in south-eastern Australia. We compiled a dataset of wildfires that destroyed houses to determine which ignition causes are more likely to result in destroyed houses and whether there are associated weather conditions that increase the probability of a destroyed house.

## Methods

**Fig 1:**
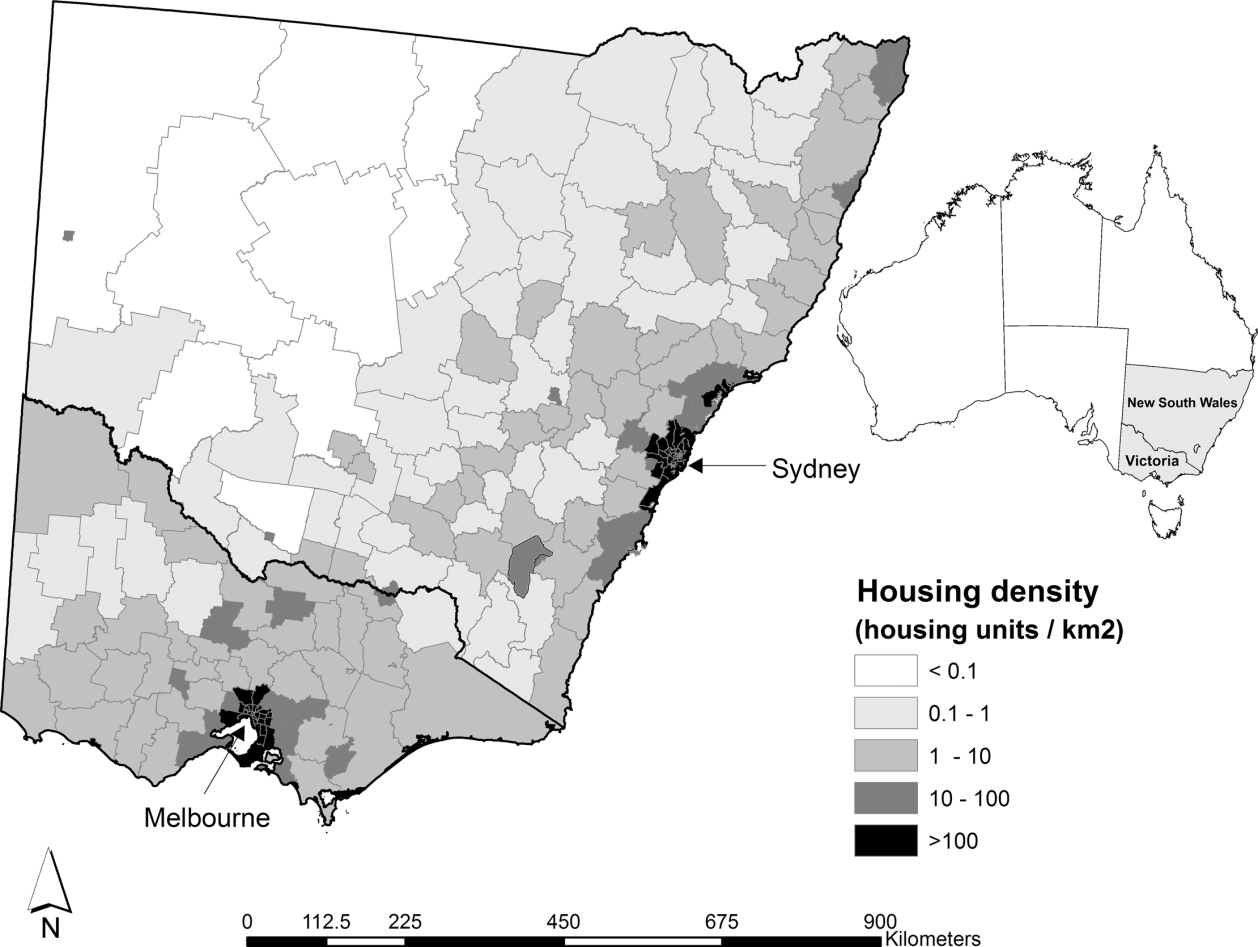
Location of study area and housing density, housing units/km^2^ in relation to local government areas. Source: generated from data from the Australian Bureau of Statistics 2011 Census of Population and Housing. Developed using Administrative Boundaries produced by PSMA Australia Limited licensed by the Commonwealth of Australia under Creative Commons Attribution 4.0 International licence (CC BY 4.0).

The study area (Fig 1) was defined by the boundaries of the states of New South Wales and Victoria. These states have the highest number of wildfires that destroyed houses in Australia [34]. Housing density is highest in Sydney and Melbourne, where two thirds of the population in the study area reside (Fig 1). Other high housing density areas are in coastal areas and a few inland cities. The major vegetation in the coastal and mountainous hinterland areas are *Eucalyptus* species dominated forests and woodlands [49, 50]. These forests can burn at very high intensities (> 50,000 kW/m) but usually with low frequency (20–100 year) [51]. Similarly the mallee eucalypts in north-western Victoria and south-western New South Wales can burn at high intensities (10,000–50,000 kW/m) also with low frequency (20–100 year) [51]. Most of the other areas are either pasture, croplands or shrublands that burn at lower intensities (< 5,000 kW/m) with frequency intervals between 5–100 years. [51].

### Long term destroyed house data

A dataset of wildfires that destroyed houses was developed by collating available data on such wildfires from July 1951 to June 2015 and their ignition cause. Although houses were destroyed by wildfire in the study area prior to 1951, most notably in 1926, 1939 and 1944 when over 500 houses were destroyed by wildfires each year [52], the available data on these wildfires was not of sufficient detail to be included. Only wildfires that destroyed a house were included in the dataset. Wildfires that only damaged houses or destroyed other buildings or property such as sheds, business premises, caravans and cars were not included in the dataset as information on these wildfires was not consistently available.

A range of information about each wildfire that destroyed a house was captured: fire name or locality, fire start date, likely date the first house was destroyed, location, number of houses destroyed, ignition cause, fire size, and fuel type. The location was recorded as the local government area where the house was destroyed as this was the finest scale the destroyed house data could be attributed to with reasonable precision. The fire size was recorded as the total number of hectares burnt by the wildfire. If multiple wildfires with the same ignition cause merged then this was recorded as a single wildfire for this cause. If fires with different ignition causes merged, then the total fire size was allocated on an equal basis for each ignition cause. Where possible, the fuel type the fire burnt through was recorded to provide an indication of fire behaviour.

A number of different data sources were accessed in order to compile the destroyed houses dataset. These included fire agency databases, annual reports and media releases, coronial inquest reports, royal commission reports, post fire review reports, Victorian municipal fire management plans, journal articles, books and newspaper articles. The details of the sources of information are provided in S1 Table. There may be additional wildfires where houses were destroyed within the study period (1951–2015) but there was insufficient information to include them in the dataset.

### 12 year comparative data

To enable a comparison of wildfires that destroyed houses and those that did not (i.e. wildfires where no houses were destroyed), wildfire ignition records were obtained from the Country Fire Authority and the Department of Environment, Land, Water and Planning in Victoria and the New South Wales Rural Fire Service. The ignition cause and date of ignition were used in the analysis. The Victorian wildfires where no houses were destroyed data included records for 12 fire years (July to June) between 1997/98 and 2008/09 and were compared against wildfires that destroyed houses in Victoria from 1997/98 to 2008/09. The New South Wales wildfires where no houses were destroyed data included records for 12 fire years between 2001/02 and 2012/13 and were compared against wildfires that destroyed houses in New South Wales from 2001/02 to 2012/13. Only wildfires that destroyed houses within the relevant 12 year period were used in the comparative analysis as the distribution of ignitions is unlikely to be same across all years of the 64 year destroyed house dataset.

Weather records from the nearest available Bureau of Meteorology station were sourced for the 12 year comparative analysis for both wildfires that did and did not destroy houses. For the day of ignition we extracted the 1500h temperature, relative humidity (RH), wind speed and calculated the forest fire danger index (FFDI). The FFDI is related to the chance of a fire igniting, its rate of spread and difficulty of suppression [53] and has been used to examine the risk of wildfires destroying houses [15, 34]. For most of the wildfires, the time of ignition was not known, so the 1500h weather was chosen as this is usually when the maximum FFDI is likely to occur [54].

Ignitions with known causes were grouped into four causal categories: deliberate, lightning, powerlines and other known (Table 1). Arson and suspicious causes were combined because wildfires that destroy houses usually undergo a detailed causal investigation that may result in more ignitions designated as arson than suspicious. The other known category could not be split any further due to the low numbers of wildfires that destroyed houses for the separate causes within the 12 year comparative period.

**Table 1:** Description of cause categories used for wildfire ignitions in the 12 year period.

Cause	Examples of fire causes within category
Deliberate	Fires where there is evidence of deliberately ignited fires, including fires ignited by juveniles and fires ignited without a fire permit i.e. illegal fires Suspicious fires where circumstances indicate that the fire was likely to be deliberately ignited but ignition source may not be identified
Lightning	Fires that result from a lightning strike
Powerlines	Fires caused by powerlines clashing, arcing or a branch or animal contacting live parts of the network or breakage of wires, poles, cross-arms, insulators or other components
Other known	Fires caused by equipment or machinery use or malfunction. Accidental escapes from prescribed burns, agricultural burns, debris burning, campfires or cooking fires. Fires accidently ignited by a cigarette or other smoking material. Fires accidently caused by ordnance training activities. Fires identified as accidental but no further details available

### Analysis

#### Long term destroyed house data

Fire sizes of lightning-caused wildfires that destroyed houses were compared to human-caused wildfires that destroyed houses using Welch’s anova. This test was chosen as the results of Bartlett’s test revealed that the data were heteroscedastic. Prior to analysis, the fire size data were checked for normality using histograms and, as the data were highly skewed, it was transformed using natural logarithms.

#### 12 year comparative data

The 12 year data of wildfires that destroyed houses and wildfires where no houses were destroyed were compared graphically by ignition cause (all causes included undetermined ignitions; deliberate, lightning, powerlines and other known) and fire weather element on the day of ignition (FFDI, temperature, wind speed and RH). The cumulative % distribution for wildfires that did and did not destroy houses in the 12 year period for each ignition cause and fire weather element was calculated. Welch’s anova was used to determine if there was a statistically significant difference between the wildfires that destroyed houses and wildfires where no houses were destroyed for each ignition cause and fire weather element. Each of the 4 known ignition causes were tested separately for each fire weather element. For example, temperature on day of ignition for powerline-caused wildfires that destroyed houses were compared to the temperature on day of ignition for the powerline-caused wildfires where no houses were destroyed. Prior to analysis, each set of data were checked for normality using histograms and a natural logarithmic transformation was applied to the FFDI data. As Bartlett tests showed that for some data the variances were not equal, Welch’s anova was chosen to compare the data. The Fisher’s exact test of independence was used to examine whether the proportion of each of the known ignition cause categories are different when compared between the wildfires that destroyed houses and wildfires where no houses were destroyed for the 12 year period. The tests were conducted using R statistical package v3.1.0 [55].

## Results

### Long term destroyed house data

From July 1951 to June 2015 there were 250 wildfires that destroyed houses, 155 where the ignition cause was identified and 95 where the cause was undetermined (Table 2). There were 7430 houses destroyed by wildfires in the 64 year study period (Table 2), with over 85% of these houses destroyed in forest fires. A third of the houses destroyed were the result of wildfires started by powerlines, 25% from fires with an undetermined cause, 22% from deliberately ignited fires and 11% from fires started by lightning strikes. The main ignition causes in the other known category were equipment / machinery use (14 wildfires, 250 houses destroyed), escapes from fuel reduction burning and agricultural burning activities (13 wildfires, 279 houses destroyed) and wildfires accidently ignited by a cigarette or other smoking material (5 wildfires, 33 houses destroyed).

**Table 2:** The number of wildfires that destroyed houses and the number of houses destroyed from 1951 to 2015 by ignition cause.

Ignition cause	No. of wildfires that destroyed houses	No. of houses destroyed
Deliberate	61	1663
Powerlines	30	2513
Lightning	29	843
Other known	35	580
Undetermined	95	1831
**Total**	**250**	**7430**

**Fig 2:**
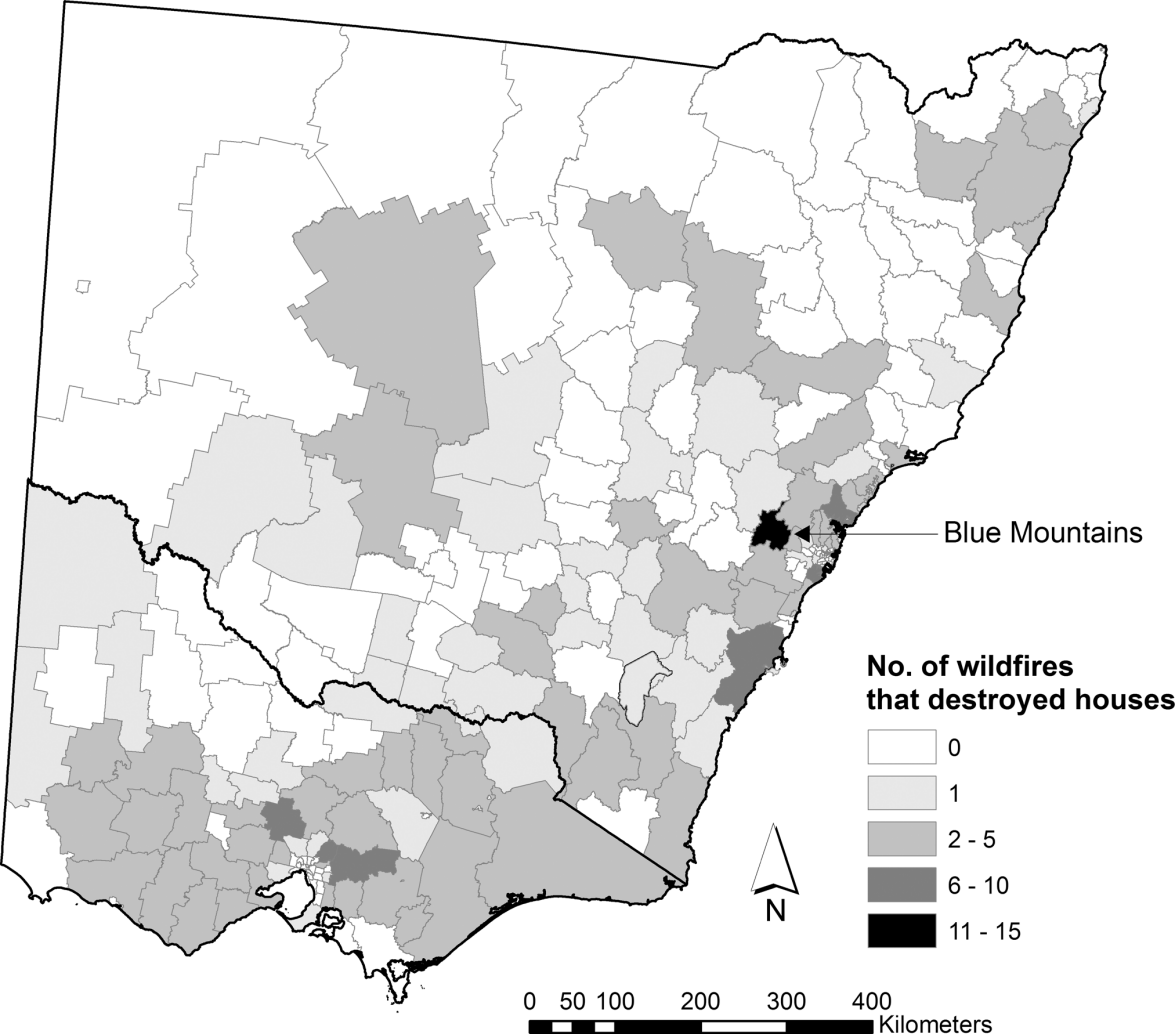
The number of wildfires that destroyed houses from 1951 to 2015 by local government area. Developed using Administrative Boundaries produced by PSMA Australia Limited licensed by the Commonwealth of Australia under Creative Commons Attribution 4.0 International licence (CC BY 4.0).

**Fig 3:**
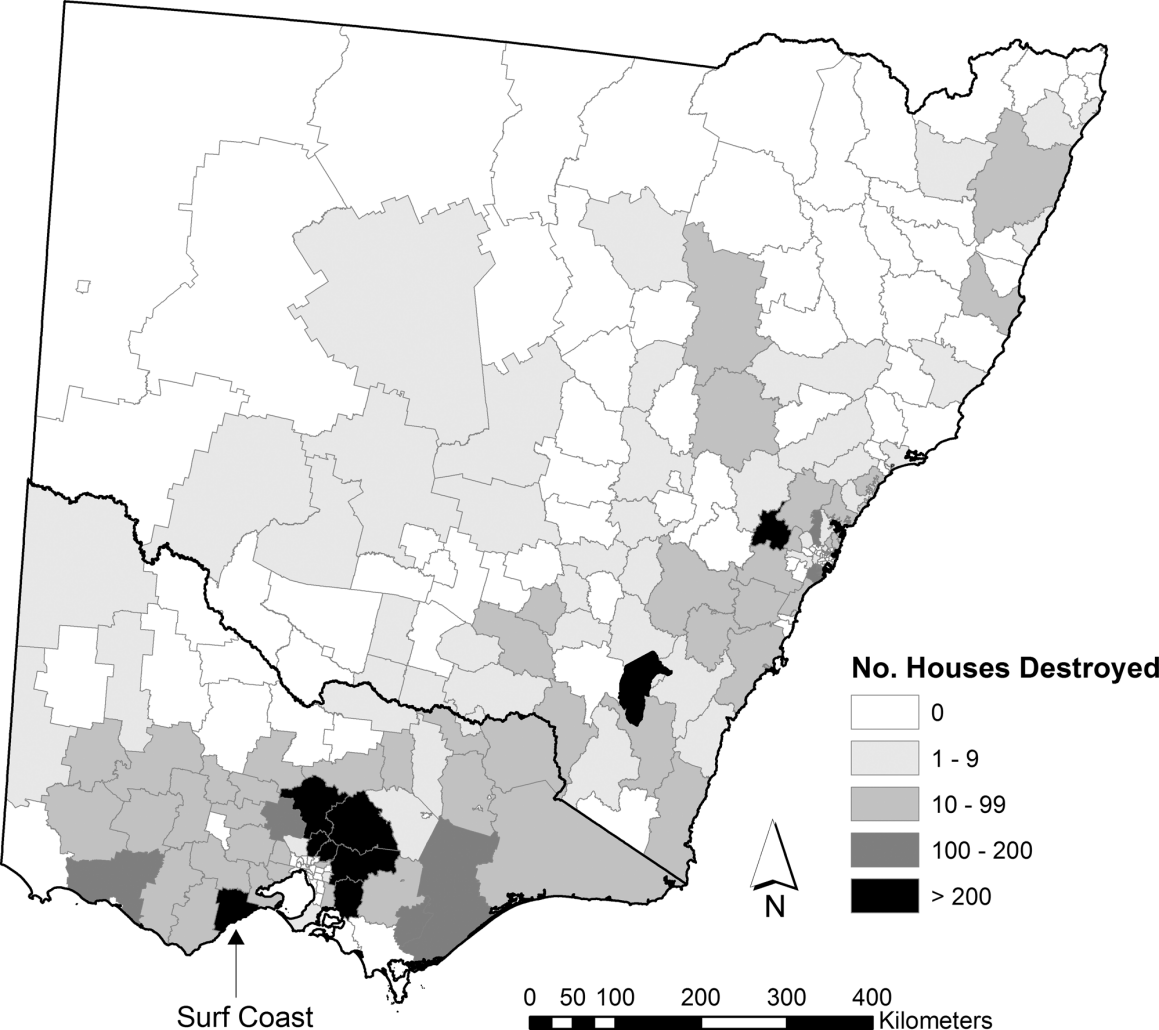
The number of houses destroyed by wildfires from 1951 to 2015 by local government area. Developed using Administrative Boundaries produced by PSMA Australia Limited licensed by the Commonwealth of Australia under Creative Commons Attribution 4.0 International licence (CC BY 4.0).

The Blue Mountains local government area, located approximately 50 km west of Sydney, had the highest number of wildfires that destroyed houses for a local government area with 15 wildfires (Fig 2). The Surf Coast local government area, located approximately 120 km southwest of Melbourne, had the highest number of houses destroyed for a local government area with 733 (Fig 3); almost all (730) were destroyed in a wildfire in 1983. Wildfires that destroy a very large number of houses in a single event are infrequent, only 6 wildfires destroyed > 200 houses. These 6 wildfires account for 48% of the total number of houses destroyed by wildfire. Over 60% of wildfires had < 10 houses destroyed in the event.

The area burnt by a wildfire that destroyed houses ranged from 2 ha to 1.15 million ha (Table 3). Lightning-caused wildfires that destroyed houses were significantly larger (*P* < 0.001) in area than human-caused wildfires: median value for lightning-caused ignitions was 26314 ha compared with 3222 ha for human-caused wildfires that destroyed houses.

**Table 3:** The number of wildfires that destroyed houses from 1951 to 2015 classified by ignition cause and fire size (ha).

Ignition cause	No. of wildfires that destroyed houses by fire size (ha)							
	< 100	100–999	1000–4999	5000–9999	10000–49999	50000–100000	> 100000	Unknown
Deliberate	4	12	18	10	9	5	1	2
Powerlines	2	6	8	1	12	1		
Lightning		2	2	6	7	4	8	
Other known	6	6	6	3	7	3	3	1
Undetermined	5	8	9	8	20	3	4	38
**Total**	**17**	**34**	**43**	**28**	**55**	**16**	**16**	**41**

The first house destroyed most often occurred on the day the wildfire started (Table 4). For wildfires started by powerlines, the first house destroyed always occurred on the day the fire started. In contrast, only 6 of 27 lightning-caused wildfires incurred a house destroyed on the day of ignition. For 10 wildfires (5 lightning-caused), it was at least 2 weeks after the fire initially started until the first house was destroyed.

**Table 4:** The number of wildfires that destroyed houses from 1951 to 2015 classified by ignition cause and the number of days from fire ignition until the first house was destroyed.

Ignition cause	No. of days from fire ignition until first house destroyed						
	0	1	2	3	5	> 5	unknown
Deliberate	50	4	1			4	2
Powerlines	30						
Lightning	6	5	2	3	3	8	2
Other known	21	3	5			3	3
Undetermined	36	2	3	1	1	2	50
**Total**	**143**	**14**	**11**	**4**	**4**	**17**	**57**

### 12 year comparative data

**Fig 4:**
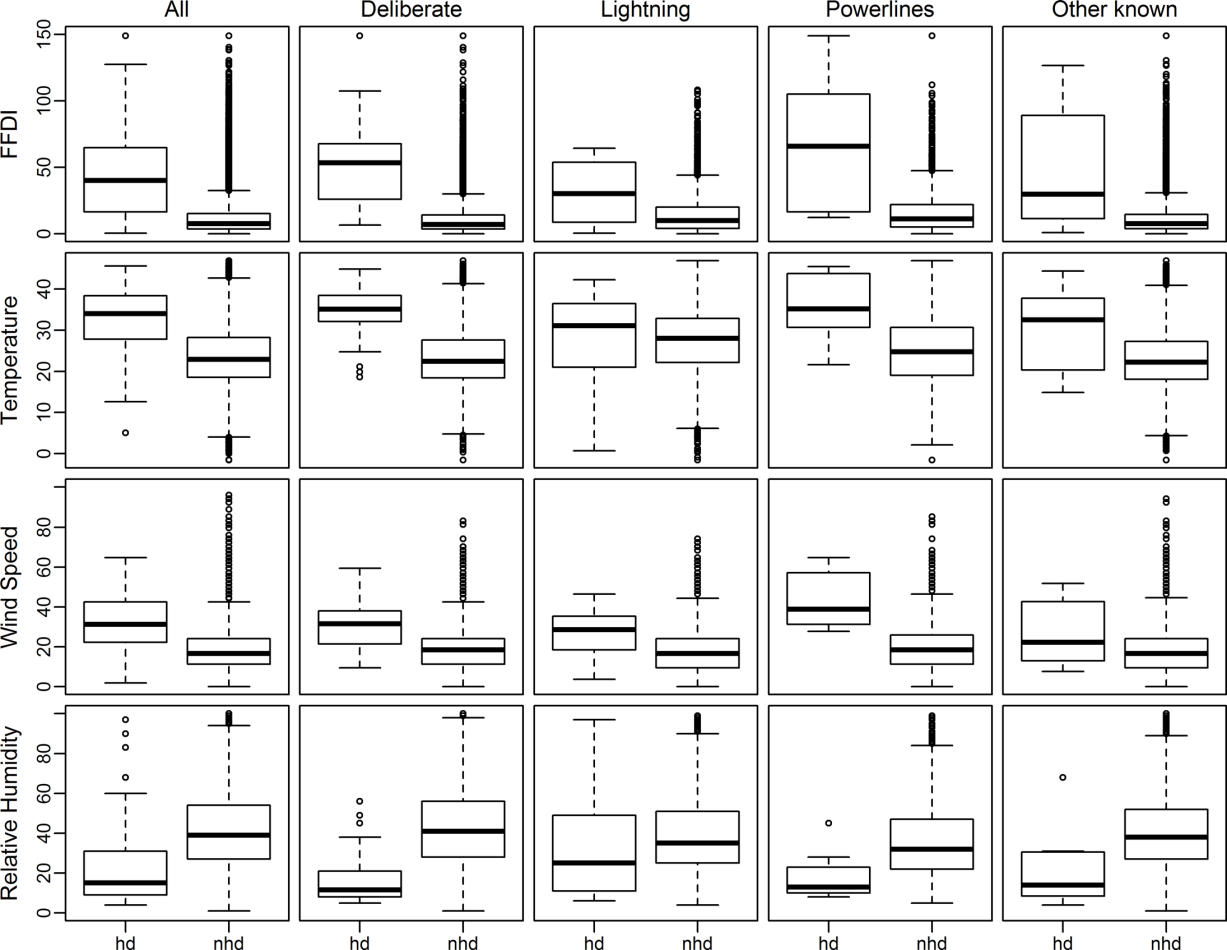
Box plots for ignition causes of wildfires that destroyed houses and wildfires where no houses were destroyed for the 12 years with complementary data for fire weather elements. Forest Fire Danger Index (FFDI), hd = wildfires that destroyed houses, nhd = wildfires where no houses were destroyed, All = all ignition causes including undetermined ignitions.

**Fig 5:**
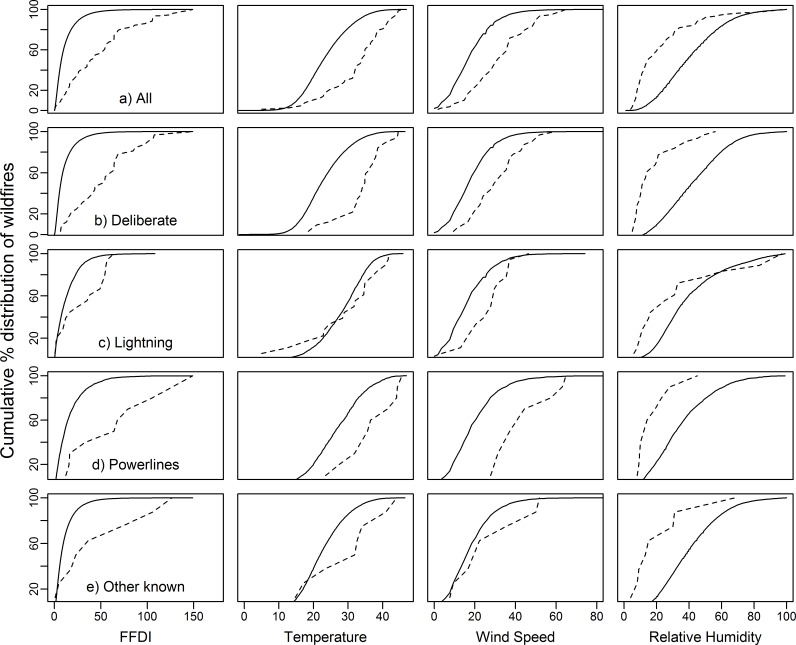
Cumulative % distribution of wildfires that destroyed houses and wildfires where no houses were destroyed by ignition cause for the 12 years with complementary data for fire weather elements. Forest Fire Danger Index (FFDI), dotted line = wildfires that destroyed houses, solid lines = wildfires where no houses were destroyed, All = all ignition causes including undetermined ignitions

For deliberate- and powerline-caused wildfires, temperature, wind speed, and FFDI were all significantly higher and RH significantly lower (*P* < 0.05) on the day of ignition for wildfires that destroyed houses compared with wildfires where no houses were destroyed in the same 12 year period (Fig 4). Lightning-caused ignitions had significantly higher wind speed (*P* < 0.05) for wildfires that destroyed houses but FFDI (*P* = 0.07), RH (*P* = 0.40) and temperature (*P* = 0.71) were not significantly different from wildfires where no houses were destroyed in the 12 year period. However, the first house was destroyed on the day of ignition for only 3 of the 18 lightning-caused wildfires in the 12 year period. The other known-caused ignitions had significantly lower RH (*P* = 0.05) for wildfires that destroyed houses but FFDI (*P* = 0.10), temperature (*P* = 0.10) and wind speed (*P* = 0.20) were not significantly different from wildfires where no houses were destroyed in the 12 year period. Most deliberate-caused wildfires that destroyed houses started when the temperature > 30°C, wind speed > 20 km/hr, RH < 25% and FFDI > 25 (Fig 5). Most powerline-caused wildfires that destroyed houses occurred when the temperature > 25°C, wind speed > 30 km/hr, RH < 25% and FFDI > 30 (Fig 5).

**Fig 6:**
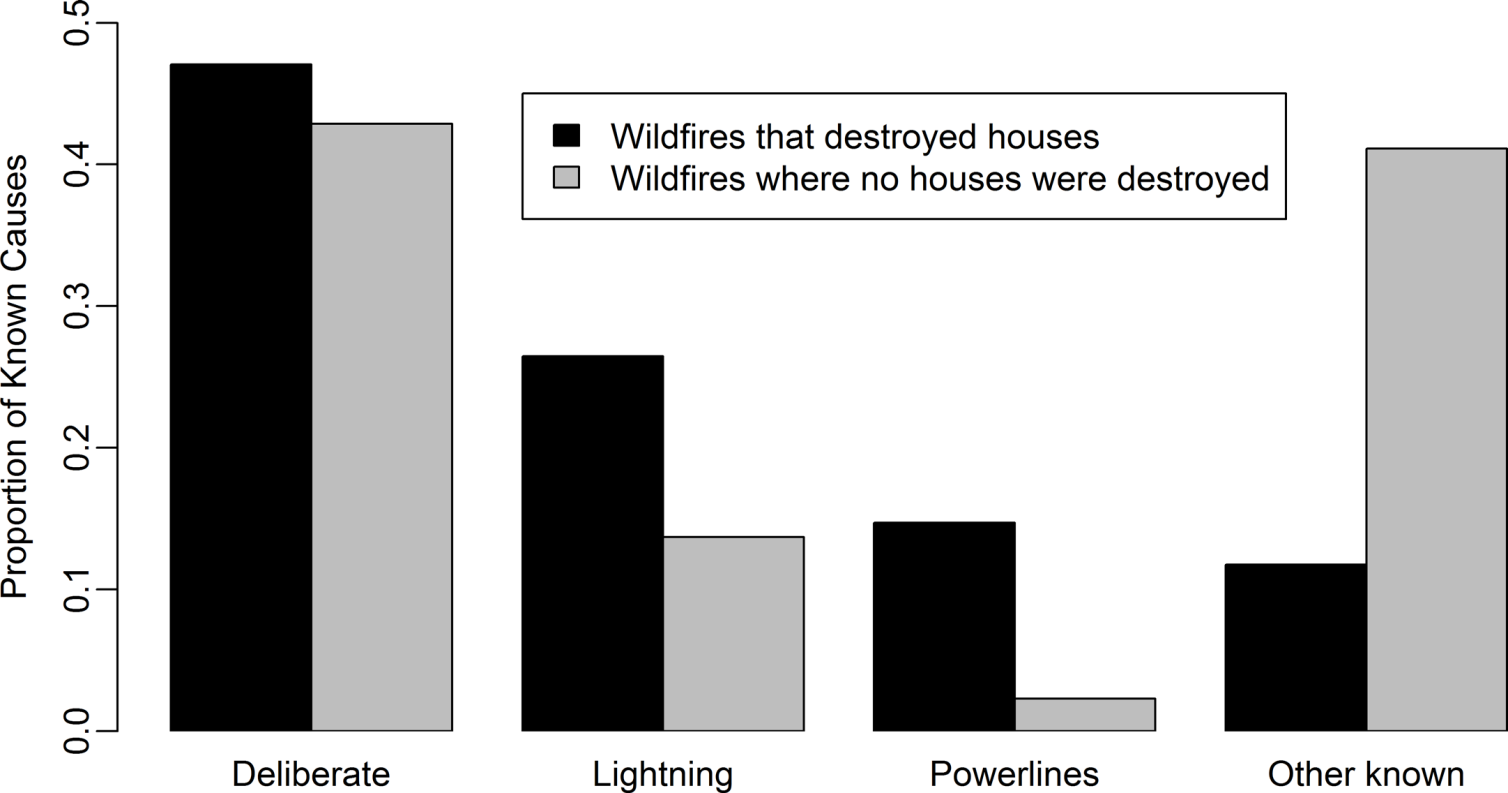
The proportion of wildfires that destroyed houses (n = 58) and wildfires where no houses were destroyed (n = 87055) by known cause for the 12 years with complementary data.

Wildfires that destroy houses are rare events with only 0.06% of wildfires resulting in a house destroyed in the 12 year comparative period. For the 12 year period, there was a significant difference in the proportion of known ignition causes for wildfires that destroyed houses (*P* < 0.001) when compared with wildfires where no houses were destroyed. Powerlines were 6 times more common in the wildfires that destroyed houses data than in the wildfires where no houses were destroyed data and lightning 2 times more common (Fig 6). The proportion of deliberate ignitions was slightly higher for wildfires that destroyed houses and other known ignitions were 3 times lower in the wildfires that destroyed houses data than the wildfires where no houses were destroyed data.

## Discussion

We found that powerlines, lightning strikes and deliberate ignitions are the main ignition causes of wildfires that destroyed houses (Table 2). Arson and powerlines are also among the main ignition causes of wildfires that destroyed houses in California [56]. For deliberate- and powerline-caused wildfires, the fire weather was significantly worse on the day of ignition for wildfires that destroyed houses compared with wildfires where houses were not destroyed (Fig 5). For deliberate ignitions, the first house destroyed most often occurred on the day of ignition whereas for powerline-caused wildfires the first house destroyed always occurred on the day of ignition (Table 4), this has not been previously reported in other studies. Our results are consistent with previous research that showed that weather and the proximity of ignition to houses are important factors in determining the probability of houses destroyed by wildfires [37, 48]. However, for lightning-caused wildfires proximity of ignition to houses may be less important as the first house destroyed from a lightning-caused wildfire most often occurred at least two days after the fire started (Table 4). For these events, weather on subsequent days after ignition is likely to be important, although houses destroyed from grass fires started by lightning strikes usually occurred within a day of the fire starting.

The proportion of deliberately ignited wildfires that destroyed houses is similar to the proportion of deliberately ignited wildfires where no houses were destroyed but powerline- and lightning-caused fires are disproportionately higher for wildfires that destroyed houses (Fig 6). While there are no similar studies investigating ignition causes and destroyed houses, the proportion of powerline-caused wildfires substantially increases in Southern California under high wind conditions and several large destructive wildfires in October 2007 were ignited by powerlines [57]. These results suggest that to decrease the number of wildfires that destroy houses, efforts should be focussed on improving the safety of powerlines, reducing the fire spread of lightning-caused wildfires and reducing the number of deliberate wildfire ignitions.

Powerline-caused ignitions were the most over-represented cause in the wildfires that destroyed houses data and resulted in the most houses destroyed. It has long been recognised that powerlines are a potential source of destructive wildfires and require actions to reduce the risk of ignitions. Inquiries following destructive wildfires in Victoria, recommended improving inspection and maintenance of powerlines and the surrounding vegetation [1, 58], improving safety equipment on networks, for example fitting spreaders to stop conductors from clashing [1, 58], installing devices that automatically switch off power when a fault occurs and changing settings on high fire risk days to reduce energy release if a fault occurs [1] and burying cables underground in high risk areas [1, 58, 59]. Following the Black Saturday fires, the Victorian government allocated $750 million to reduce the risk of powerlines causing wildfires, including $200 million to replace network and private powerlines in the highest risk wildfire areas and $500 million to electricity network operators to install new technologies that will better control the faults that may cause fires [60]. Additionally, regulations have been strengthened with major network operators required to prepare a bushfire mitigation plan that details how the network operator will minimise the risk of fire ignition from its supply network and report annually of its performance to an independent regulator. The plans are independently audited and the regulator can direct network operators to implement or modify their plans. If private powerlines are not maintained, then there are provisions to enable network operators to enter the land and undertake the work. For example, in Victoria, the *Electricity Safety Act 1998* and *Electricity Safety (Bushfire Mitigation) Regulations 2013* detail the plan requirements and schedules for inspecting, testing, maintaining and upgrading network assets. The *Electricity Safety (Electric Line Clearance) Regulations 2015* mandates the minimum vegetation clearance distances for overhead powerlines in Victoria and requires network operators to submit an annual plan for vegetation clearance for approval. Similarly, Californian regulations were strengthened after destructive wildfires caused by powerlines in Southern California in 2007 [61, 62].

Destroyed houses from powerline-caused wildfires may be largely prevented if the power is temporarily shut off on high fire risk days. There are legislative arrangements that provide for this but they are considered a last resort option as the potential impact on the community may outweigh the risk of leaving the power in service [58, 61, 63]. Temporarily shutting off the power on high fire risk days will also impact on communication networks important for issuing fire warnings to the community, may disrupt water supply and adversely affect the welfare of vulnerable community members. Alternatively, burying cables underground will also eliminate the fire risk but this is expensive e.g. $40 billion for rural areas in Victoria [63]. To date, other measures have been preferred, but it is not yet known whether investing in new technologies, upgrading networks and adopting stricter standards on the design, inspection and maintenance of networks will substantially reduce the potential for powerline-caused destructive wildfires. However, if powerlines are found to be the ignition source of a destructive wildfire, then it is highly likely that network operators will face substantial claims for damages and compensation. Litigation following the Black Saturday fires has seen electricity network operators required to pay over $700 million in damages to people who suffered losses in the fires [64–67].

Lightning-caused wildfires that destroyed houses were found to be significantly larger in size than human-caused wildfires that destroyed houses. This result can be explained by the spatial patterns of ignitions as lightning ignitions typically occur further away from houses than human caused ignitions [29, 47, 68] and take longer to reach houses. Their remoteness from populated places may limit fire suppression efforts due to lengthy response times for resources to reach the wildfire. Prevention of lightning is of course impossible but fuel reduction treatments may reduce fires spreading from lightning strikes [37, 69] and improve the probability of successful fire control [17]. These treatments are most effective if a wildfire encounters them within 5 years of treatment [70, 71] but under adverse fire weather conditions the fire intensity may still be too high for safe and effective fire suppression [27] and most houses are destroyed when the FFDI > 50 [34]. Landscape fuel reduction treatments where lightning occurs may be ineffective in limiting the fire spread toward the interface as the level of treatment required to substantially alter the risk of wildfires destroying houses is very large [40].

Deliberate ignitions typically occur in easily accessible areas, close to urban centres [29, 44, 72]. Unlike other ignition causes, the arsonist chooses the timing and location. When these ignitions result in destructive consequences pressure is often placed on governments, land managers, fire and law enforcement agencies to reduce arson ignitions [73]. In response, severe penalty provisions for arson offences have been enacted in Australian, United States and Mediterranean jurisdictions although there is no clear evidence to suggest that this deters arsonists [73, 74]. However, the fear of being caught may deter arsonists [75] and a recent study has shown increasing the number of law enforcement officers led to a decrease in deliberately ignited fires [76]. Preventing deliberate ignitions is difficult as there will always be some people who choose to light wildfires [73] and arsonists are rarely caught [74, 77]. There is limited knowledge on why and how often people light fires [78]; what is known is based on those who have been caught and may not be representative of the those who avoid apprehension [73, 78]. As a consequence, reducing deliberate wildfire ignitions is likely to be more successful if strategies are concentrated on where fires are ignited (arson hot-spots) rather than the profile of an arsonist [77]. Potential prevention strategies for arson hots spots include: community education and arson awareness programmes; reducing fuels in the area; limiting access and increasing patrols of these areas on days of very high fire danger [77]. It is difficult to evaluate how effective these strategies are as changes in the number of ignitions need to be considered in the context of variations in fire weather and fuel availability over time. However, a Western Australia study has correlated the reduction in the number of deliberate ignitions [79] to a targeted arson reduction programme in the area [80].

Many of the other known ignitions occur due to the careless use of fire or equipment/machinery. Laws have been enacted to reduce these types of ignitions, by restricting when and how activities that may cause wildfires are conducted. For example, machinery such as tractors and harvesters must be fitted with a spark arrester and carry fire suppression equipment. Permits are required to light a fire, except for a cooking fire, in the open during the fire danger period. The fire danger period is typically declared for several months at the onset of warmer weather and when the vegetation becomes drier. A total fire ban may be declared (usually for a 24 h period) when predicted fire behaviour indicates wildfires are likely to spread rapidly and be difficult to control (typically when the FFDI > 50). A total fire ban prohibits the lighting of fires in the open and the use of hot works equipment, such as welding or grinding. These laws will only be effective if people know and understand them. Investigations following an equipment-caused wildfire that destroyed houses in Western Australia found 33% of people interviewed were not aware that a total fire ban had been declared [81] and there was a lack of understanding of what activities were prohibited [82].

Our study was limited because 38% of wildfires that destroyed houses the ignition cause was undetermined. In recent years, improvements in fire agency record keeping, the availability of fire investigation specialists and technology such as lightning strike detection systems, has resulted in increased reliability and quality of data on ignition causes.

Improving powerline safety and targeted arson reduction programmes may reduce some wildfire ignitions but there is still potential for houses to be destroyed by wildfires, particularly during extreme weather conditions. Fuel management and suppression resources may reduce fire spread but these are most effective under more benign weather conditions [27, 37]. Containment success is more likely when suppression resources reach the fire when it is small in size [16, 17, 83]. The early detection of ignitions and the placement of resources in strategic locations to minimise response time [84] may improve suppression effectiveness. Other measures are centred around increasing the resilience of houses to wildfire impacts, e.g. reducing the exposure of houses to wildfire attack by development planning and building controls, and educating residents on preparing their property for wildfire. Land use and zoning measures can be used to prevent housing developments from occurring in wildfire prone areas or require houses to comply with building construction standards and fire protection measures [9, 85, 86]. Designing or retrofitting houses to prevent ember penetration will improve the chance of a house’s survival in a wildfire as embers are the predominant mechanism of house ignitions from wildfires [21, 86, 87]. Reducing potential radiant heat and flame exposure can be achieved by siting the house relative to flammable vegetation and building construction standards [21, 86, 87]. House survival from a wildfire is more likely if the vegetation in a 40m zone surrounding a house is well maintained and there are no combustible objects within this zone [21, 28, 87]. Active defence of the house will also increase its chance of survival [20, 24, 87] although residents must be well prepared both physically and mentally if they are to undertake fire suppression activities [88].

Our study has highlighted the major wildfire ignition causes that result in destroyed houses, however focussing on this area only, will not reap the greatest reduction in houses destroyed by wildfires. A combination of fire management, planning and resident actions is required to reduce the number of houses destroyed by wildfires.

## Supporting Information

S1 Table Sources of information for wildfires that destroyed houses. (DOCX)
