# Supplementary material for: Some Wildfire Ignition Causes Pose More Risk of Destroying Houses than Others
Source: PLoS One. 2016 Sep 6;11(9):e0162083. doi: 10.1371/journal.pone.0162083 (PMC5012638; doi:10.1371/journal.pone.0162083)
Supplement: S1 Table — (DOCX) [file pone.0162083.s001.docx]

**S1 Table. Sources of information for wildfires that destroyed houses.**

| **Ref. No.** | **Source** |
| --- | --- |
| 1 | Barber, EHE (1977) Report of the Board of Inquiry into the Occurrence of Bush and Grass fires in Victoria. Victorian Legislative Assembly. (Government Printer: Melbourne) |
| 2 | Benalla Rural City Council Municipal Fire Management Plan Review December 2013 |
| 3 | Bence B (1989) ‘Fire: the story of a community’s fight against fire’, (NB Bence, Warrandyte, Victoria) |
| 4 | Blanchi, R, Lucas, C, Leonard, J, Finkele, K (2010) Meteorological conditions and wildfire-related house loss in Australia. *International Journal of Wildland Fire* **19**, 914-926. |
| 5 | Chatto K, Tolhurst K, Leggett A, Treloar A (1999) Development, behaviour, threat and meteorological aspects of a plume driven bushfire in west-central Victoria: Berringa fire, February 25-26 1995, Dept. of Natural Resources & Environment, Fire Research Report No. 48 (East Melbourne, Victoria) |
| 6 | Cheney NP (1976) Bushfire disasters in Australia, 1945-1975, *Australian Forestry* **39,** 245-268. |
| 7 | Clode D (2010) ‘A future in flames’, (Melbourne University Press, Carlton, Victoria) |
| 8 | Collins P (2006) ‘Burn: the epic story of bushfire in Australia’, (Allen & Unwin, Crows Nest, NSW) |
| 9 | Collyer R (2002) Incident Controller Report, Richmond Valley Rural Fire District Section 44 declaration, December 24^th^ 2001 to January 3^rd^ 2002, Serendipity Fire, New South Wales Rural Fire Service |
| 10 | Condon RW (1975) Report on bushfires in the Western Division of New South Wales, November 1974 to March 1975 |
| 11 | Cotterill R (2002) Section 44 report, Penrith / Blacktown / Liverpool / Fairfield, 25 – 30 December 2001, New South Wales Rural Fire Service |
| 12 | Country Fire Authority Annual Report 2007 |
| 13 | Country Fire Authority major fires website <http://www.cfa.vic.gov.au/about/major-fires/> |
| 14 | Country Fire Authority news 3/2/2012, Major Wodonga fire: 60 years on, <http://54.206.64.143/news/major-wodonga-fire-60-years-on.html> |
| 15 | Cunningham CJ (1984) Recurring natural fire hazards: a case study of the Blue Mountains, New South Wales, Australia, *Applied Geography* **4,** 5-27. |
| 16 | Department of Environment and Primary Industries ignitions database |
| 17 | Department of Environment and Primary Industries major fires website <http://www.depi.vic.gov.au/fire-and-emergencies/managing-risk-and-learning-about-managing-fire/bushfire-history> |
| 18 | Duggin JA (1976) Bushfire history of the south coast study area, CSIRO Division of Land Use Research, Technical memorandum 76/13 |
| 19 | Emergency Management Victoria (2015) 2013/2014 Fire season: overview and narrative, Victorian Government, Melbourne |
| 20 | Esplin B, Gill M, Enright N (2003) Report of the inquiry into the 2002-2003 Victorian bushfires, State Government of Victoria |
| 21 | Fire Refuge Review Working Party (2001) Fire refuge review: examining the role of fire refuges in helping people minimise the risks of bushfire in Victoria, Victorian Emergency Management Council |
| 22 | Forests Commission Victoria Annual Report 1982-83 |
| 23 | Hiatt J (1992) Inquest into the death of Shirley Anne Dudley, Inquest into the death of Emma Selina Tracy Burns, Fire Inquiry concerning fire at 13 Orana Road Kenthurst, Coroner’s Court Westmead, New South Wales |
| 24 | Hiatt J (1994) New South Wales Bushfire Inquiry, Coroner’s Court Westmead, New South Wales |
| 25 | Johnstone G (1998) 1997 Dandenong Ranges fires: inquests into the deaths of Jennifer Louise Lindroth, Graham Kingsley Lindroth and Genevieve Erin during a fire at Ferny Creek and four other fires in the Dandenong Ranges on 21^st^ January 1997, State Coroner’s Office Victoria |
| 26 | Johnstone G (2002) Report of the investigation and inquests into a wildfire and the deaths of five firefighters at Linton on 2 December 1998, State Coroner’s Office Victoria |
| 27 | Lane D (2002) Section 44 report, Appin Road and Burke Road Fires, 25 December 2001 to 14 January 2002, New South Wales Rural Fire Service |
| 28 | Luke RH, Koperberg PC, Grady K, Tolhurst J (1978) Report on various aspects relating to property damage, 16^th^, 17^th^ and 18^th^ December, 1977 Blue Mountains Bush Fires, Blue Mountains Fire Prevention Association. |
| 29 | Luke, RH, McArthur, AG (1978) ‘Bushfire in Australia.’ (Australian Government Publishing Service: Canberra) |
| 30 | Malua Bay Rural Fire Brigade website, <http://www.maluabay.rfsa.org.au/Fire_History.htm> |
| 31 | Maurice Blackburn Lawyers website <http://www.mauriceblackburn.com.au/legal-services/general-law/class-actions/current-class-actions/bushfire-class-actions/murrindindimarysville-bushfire-class-action/> |
| 32 | Maynes KJ and Garvey MF (1985) Report on selected major fires in country areas of Victoria on 14 January 1985, Country Fire Authority, Victoria |
| 33 | McLeod R (2003) Inquiry into the Operational Response to the January Bushfires, Australian Capital Territory Government: Canberra |
| 34 | Milovanovich C (2004) Inquest into the death of Ronald Gillett and associated fire, Coroner’s Court, East Maitland, New South Wales |
| 35 | Mornington Peninsula Municipal Fire Management Planning Committee (2013) Mornington Peninsula Municipal Fire Management Plan |
| 36 | Murray R, White K (1995) ‘State of fire: a brief history of volunteer firefighting and the Country Fire Authority in Victoria’ (Hargreen Publishing Company, North Melbourne, Victoria) |
| 37 | Murrindindi Shire and Lake Mountain Municipal Fire Management Plan 2012, Version 5.2 |
| 38 | NSW Bush Fire Committee (1965) Report of the New South Wales Bush Fire Committee for the year ended 30^th^ April 1965 (Government Printer, Sydney NSW) |
| 39 | NSW Bush Fire Committee (1969) Report of the New South Wales Bush Fire Committee for the year ended 30^th^ April 1969 (Government Printer, Sydney NSW) |
| 40 | NSW Bush Fire Committee (1970) The 1969/70 season. *Bush Fire Bulletin* **8(1)**, 1-2. |
| 41 | NSW Bush Fire Committee (1975) Tragic fire season: 1974-5. *Bush Fire Bulletin* **9(9)**, 19-20. |
| 42 | NSW Bush Fire Committee (1975) A lucky save. *Bush Fire Bulletin* **9(10)**, 1-2. |
| 43 | NSW Bush Fire Committee (1979) Late fire season in New South Wales. *Bush Fire Bulletin* **1(2)**, 3-7. |
| 44 | NSW Bush Fire Council (1975) Report of the Co-ordinating Committee of the Bush Fire Council of New South Wales for the year ended 31 March 1975 (Government Printer, Sydney NSW) |
| 45 | NSW Bush Fire Council (1980) Report of the Co-ordinating Committee of the Bush Fire Council of New South Wales for the year ended 31 March 1980 (Government Printer, Sydney NSW) |
| 46 | NSW Department of Bush Fire Services Annual Report 1989/1990 |
| 47 | NSW Department of Bush Fire Services Annual Report 1990/1991 |
| 48 | NSW Department of Bush Fire Services Annual Report 1991/1992 |
| 49 | NSW Department of Bush Fire Services (1987) Preliminary report on the 1986-87 bush fire season, *Bush Fire Bulletin* **9(1&2)**:4-5 |
| 50 | NSW Department of Bush Fire Services (1990) Early fire season in the north, *Bush Fire Bulletin* **12(3)**:24 |
| 51 | NSW Department of Bush Fire Services (1991) North coast family left homeless, *Bush Fire Bulletin* **13(2)**:23 |
| 52 | NSW National Parks and Wildlife Service (2006) Cuumbeun, Stony Creek & Wanna Wanna Nature Reserves Fire Management Strategy |
| 53 | NSW Office of Environment and Heritage fire history mapping database |
| 54 | NSW Parliamentary Debates, Legislative Assembly, 22 June 2000, page 7594, Available at <http://www.parliament.nsw.gov.au/prod/parlment/hansart.nsf/V3Key/LA20000622052> |
| 55 | NSW Parliamentary Debates, Legislative Assembly, 5 September 2002, page 4705, Available at <http://www.parliament.nsw.gov.au/prod/parlment/hansart.nsf/V3Key/LA20020905008> |
| 56 | NSW Rural Fire Service Bushfire Building Impact Assessment database |
| 57 | NSW Rural Fire Service Fire Incident Reporting System database |
| 58 | NSW Rural Fire Service Fire Investigation Unit database |
| 59 | NSW Rural Fire Service (1998) The 97/98 fire season, *Bushfire Bulletin*, **20(1)**: 8 |
| 60 | NSW Rural Fire Service (2002) Engadine (Mount Carmel fire), *Bushfire Bulletin*, **24(4)**: 12 |
| 61 | NSW Rural Fire Service (2003) 600 fight blazes in Northern Tablelands, *Bushfire Bulletin*, 25(3): 3-4 |
| 62 | NSW Rural Fire Service (2003) Touga – Shoalhaven’s second major fire emergency in twelve months, *Bushfire Bulletin*, **25(1)**: 3-4 |
| 63 | NSW Rural Fire Service (2010) Our Service’s Story, *Bush Fire Bulletin* **32(2)**:25 |
| 64 | NSW Rural Fire Service media release, 2/11/2001, <http://144.140.132.149/dsp_more_info.cfm?CON_ID=1396&CAT_ID=606> |
| 65 | NSW Rural Fire Service media release, 22/12/2001, <http://144.140.132.149/dsp_more_info.cfm?CON_ID=1422&CAT_ID=602> |
| 66 | NSW Rural Fire Service media release, 1/11/2002, <http://144.140.132.149/dsp_more_info.cfm?CON_ID=1575&CAT_ID=602> |
| 67 | NSW Rural Fire Service media release, 5/12/2002, <http://144.140.132.149/dsp_more_info.cfm?CON_ID=1644&CAT_ID=602> |
| 68 | NSW Rural Fire Service media release, 6/12/2002, <http://144.140.132.149/dsp_more_info.cfm?CON_ID=1650&CAT_ID=602> |
| 69 | Phillips D (2002) Section 44 report Wollondilly, Wingecarribee Shires and Campbelltown RFS District, 25 December 2001 – 14 January 2002, NSW Rural Fire Service |
| 70 | Poole R (2006) A recollection of events: Baulkham Hills Complex, Day 1 of the Chilvers Lane Fire December 2002, NSW Rural Fire Service Unpublished report |
| 71 | Pyrenees Shire Municipal Fire Management Plan 2012-2015 |
| 72 | Richmond Valley Council (2002) 2002 Supplementary state of the environment report, Richmond Valley Council, Casino, NSW |
| 73 | Rowville-Lysterfield Community News website, The 1973 bush fire in Lysterfield and Rowville, Available at <http://www.rlcnews.com.au/main/about-us/history/part-b/bush-fire-1973/> |
| 74 | Smith R (2006) Debrief outcomes: significant Victorian fires December 2005 and January 2006, Report for the Department of Sustainability & Environment and the Country Fire Authority, Victoria |
| 75 | Smith S (2002) Bushfires, NSW Parliamentary Library Briefing Paper No 5/02 |
| 76 | State Electricity Commission of Victoria (1977) Statistics – fires allegedly involving SEV 8/1/59 – 30/3/77, Exhibit 202 Board of Inquiry into the occurrence of bush and grass fires in Victoria. |
| 77 | Surf Coast Shire Municipal Fire Management Plan 2011 – 2014, Version 7 July 2012 |
| 78 | Teague B, McLeod R, Pascoe S (2010) 2009 Victorian Bushfires Royal Commission Final Report. Parliament of Victoria, Melbourne. |
| 79 | The ABC website <http://www.abc.net.au/news/2013-01-30/75yo-charged-over-aberfeldy-bushfire/4491376> |
| 80 | The ABC website <http://www.abc.net.au/news/2007-01-17/bushfires-threaten-properties-in-north-east-vic/2174006> |
| 81 | The Adelaide Advertiser website <http://www.adelaidenow.com.au/ipad/bushfire-heads-for-east-gippsland-towns/story-fn6bqphm-1225998341353> |
| 82 | The Age website <http://www.theage.com.au/articles/2003/01/22/1042911428905.html> |
| 83 | The Age website <http://www.theage.com.au/articles/2003/01/30/1043804465576.html> |
| 84 | The Age website <http://www.theage.com.au/articles/2006/03/13/1142098388150.html> |
| 85 | The Age website <http://www.theage.com.au/environment/weather/residents-angry-over-victorian-fire-info-20130109-2cgmx.html> |
| 86 | The Age website <http://www.theage.com.au/victoria/fire-rages-through-farm-region-20130109-2cgul.html> |
| 87 | The Age website <http://www.theage.com.au/victoria/victoria-bushfires-claim-five-homes-residents-told-to-evacuate-20141217-128rjd.html> |
| 88 | The Age website <http://www.theage.com.au/victoria/house-destroyed-residents-urged-to-leave-as-firefighters-battle-blaze-at-moyston-near-ararat-20150102-12h94l.html> |
| 89 | The Argus, 6 February 1952, Bush fires racing far and wide across state, Retrieved from <http://trove.nla.gov.au/ndp/del/article/23161363> |
| 90 | The Benalla Ensign, 7 February 1952, Country destroyed, ravaged by bushfires 11 Lurg homes burnt, Retrieved from <http://trove.nla.gov.au/ndp/del/article/65501393> |
| 91 | The Examiner, Launceston, 1 February 1952, Fire ravages town, Retrieved from <http://trove.nla.gov.au/ndp/del/article/52846120> |
| 92 | The Hills website, Hills voices on line Glenhaven, <http://www.bhsc.nsw.gov.au/external/hillsvoices/glenhaven01.htm> |
| 93 | The Sydney Morning Herald, 16 November 1951, Fires flare up again over state, homes burned near Sydney, Retrieved from <http://trove.nla.gov.au/ndp/del/article/18239614> |
| 94 | The Sydney Morning Herald, 17 November 1951, Nearly £100,000 damage in Newcastle district, Retrieved from <http://trove.nla.gov.au/ndp/del/article/18239905> |
| 95 | The Sydney Morning Herald, 19 December 1951, Bushfires menace townships, three evacuated but saved, Retrieved from <http://trove.nla.gov.au/ndp/del/article/18244900> |
| 96 | The Sydney Morning Herald, 1 February 1952, Fire fighters save Hornsby homes, Retrieved from <http://trove.nla.gov.au/ndp/del/article/18249717> |
| 97 | The Sydney Morning Herald, 2 February 1952, New fire threat in north, flames encircle hospital, Retrieved from <http://trove.nla.gov.au/ndp/del/article/18256174> |
| 98 | The Sydney Morning Herald website <http://www.smh.com.au/news/national/man-dies-homes-destroyed-as-bushfires-rage-across-three-states/2006/12/14/1165685828198.html> |
| 99 | The Sydney Morning Herald website <http://www.smh.com.au/environment/arson-fear-as-fire-destroys-homes-20130328-2gtk8.html> |
| 100 | Tocumwal website, Sydney Morning Herald 5/1/1990 ‘The day the birds piled up and fireballs soared’ Available at <http://www.tocumwal.com.au/tocumwal-articles/1990/1/5/the-day-the-birds-piled-up-and-fireballs-soared/> |
| 101 | Wamboin Community Association History website, <http://wamboincommunity.asn.au/firebrigade/index.php?op=historyChapter> |
| 102 | Wellington Municipal Fire Management Plan 2013-2016, Version 2.1, November 2014 |
| 103 | Williams M (2002) Report of the Blue Mountains Section 44, 24 December 2001 – 22 January 2002, New South Wales Rural Fire Service |
| 104 | Wilmoth W (1992) Record of investigation into a fire at Warrandyte on 25^th^ February 1991, Case No. 626/91 State Coroner Victoria, Melbourne |
| 105 | *Woodend Water v Hyan* 1990 (Victoria Supreme Court Full Court) |
